# Design, Synthesis and Anticancer Screening of Novel Benzothiazole-Piperazine-1,2,3-Triazole Hybrids

**DOI:** 10.3390/molecules23112788

**Published:** 2018-10-27

**Authors:** Mohamed R. Aouad, Moataz A. Soliman, Muath O. Alharbi, Sanaa K. Bardaweel, Pramod K. Sahu, Adeeb A. Ali, Mouslim Messali, Nadjet Rezki, Yaseen A. Al-Soud

**Affiliations:** 1 Department of Chemistry, Faculty of Science, Taibah University, Al-Madinah Al-Munawarah 30002, Saudi Arabia; moatazalsayed@hotmail.com (M.A.S.); moharbi5056@gmail.com (M.O.A.); AdeebAli@Dal.Ca (A.A.A.); aboutasnim@yahoo.fr (M.M.); nadjetrezki@yahoo.fr (N.R.); 2 Department of Pharmaceutical Sciences, School of Pharmacy, University of Jordan, Amman 11942, Jordan; S.Bardaweel@ju.edu.jo; 3 School of Study in Chemistry, Jiwaji University, Gwalior 474011, India; researchdata6@gmail.com; 4 Department of Chemistry, Faculty of Sciences, University of Sciences and Technology Mohamed Boudiaf, Laboratoire de Chimie et Electrochimie des Complexes Metalliques (LCECM) USTO-MB, P.O. Box 1505, El M‘nouar, Oran 31000, Algeria; 5 Faculty of Science, Al al-Bayt University, Al-Mafraq 25113, Jordan

**Keywords:** Benzothiazole, piperazine, 1,2,3-triazole, anticancer activity

## Abstract

A library of novel regioselective 1,4-di and 1,4,5-trisubstituted-1,2,3-triazole based benzothiazole-piperazine conjugates were designed and synthesized using the click synthesis approach in the presence and absence of the Cu(I) catalyst. Some of these 1,2,3-triazole hybrids possess in their structures different heterocyclic scaffold including 1,2,4-triazole, benzothiazole, isatin and/or benzimidazole. The newly designed 1,2,3-triazole hybrids were assessed for their antiproliferative inhibition potency against four selected human cancer cell lines (MCF7, T47D, HCT116 and Caco2). The majority of the synthesized compounds demonstrated moderate to potent activity against all the cancer cell lines examined. Further, we have established a structure activity relationship with respect to the in silico analysis of ADME (adsorption, distribution, metabolism and excretion) analysis and found good agreement with in vitro activity.

## 1. Introduction

Nitrogen containing heterocycles comprising of triazoles, benzothiazoles, benzimidazoles, indoles, etc. constitute an important scaffold in biological science and medicinal chemistry, and has fascinating applications in drug discovery and development [1,2,3,4,5]. In particular, the synthesis of 1,2,3-triazoles has attracted considerable attention during the last years. Several potent pharmacological properties such as anti-bacterial [6], antimicrobial [7], antioxidant [8], anticancer [9], and antitubecular [10] of 1,2,3-triazole derivatives have been reported. Some clinically and commercially approved drugs including Carboxyamidotriazole [11], Tazobactam [12], and Cifatrizine [13] were found to possess the 1,2,3-triazole core in their structures.

Benzothiazole derivatives become a major area of emphasis for the organic chemists due to varied spectrum of pharmacological profile for instance, antitubercular [14], antimicrobial [15], antimalarial [16], anticonvulsant [17], anthelmintic [18], analgesic [19], antidiabetic [20], and anticancer [21]. Furthermore, benzothiazole derivatives incorporating piperazine moiety have been reported to exhibit a various biological activities such as antimicrobial [22], anti-inflammatory [23], toxicological [24], and antidepressant activities [25].

Recently, a combination of benzothiazole and 1,2,3-triazole moieties has received great interest in improving the effectiveness of bioactive molecules with fascinating anticancer activity [26,27].

In continuation of our research on the synthesis and biological evaluation of benzothiazoles and 1,2,3-triazoles [28,29,30,31,32], we here report the click synthesis and antiproliferative evaluation of new series of benzothiazole-piperazine-1,2,3-triazole hybrids incorporating different functionalities and/or heterocyclic moieties on the 1,2,3-triazole ring. The newly designed hybrids have been evaluated for their anticancer activity against breast and colon cancer cell lines in order to investigate if the combination of these heterocyclic units in one scaffold could generate active pharmaceutical ingredients (API) dotted with relevant chemotherapeutic activities comparable to the clinically approved standard drugs. To support in vitro biological assay, in silico techniques have been widely used. In this research study, we briefly describe in silico ADME prediction, with an emphasis on structure pattern recognition.

## 2. Results and Discussion

### 2.1. Chemistry

**Scheme 1:**
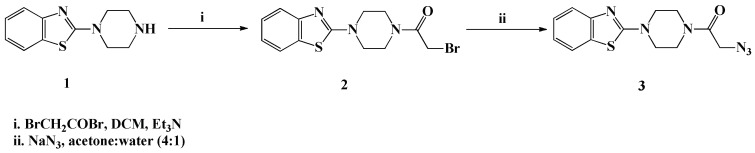
Synthesis of 2-azido-1-(4-(benzo[*d*]thiazol-2-yl)piperazin-1-yl)ethanone (**3**).

**Scheme 2:**
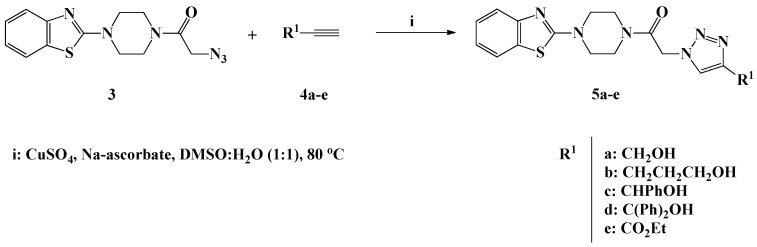
Click synthesis of 1,4-disubstituted-1,2,3-triazoles **5a**–**e** carrying functionalized alkyl side chains.

**Scheme 3:**
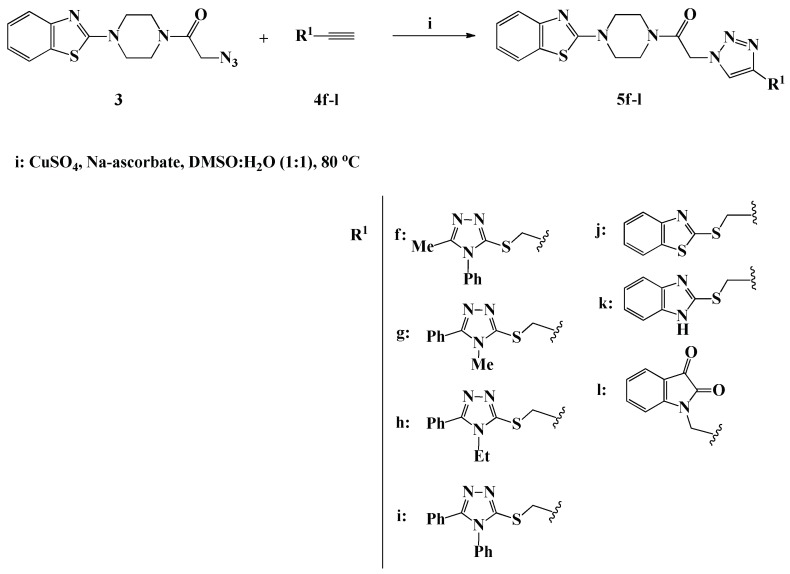
Click synthesis of 1,4-disubstituted-1,2,3-triazoles **5f**–**l** carrying heterocyclic moieties.

**Scheme 4:**
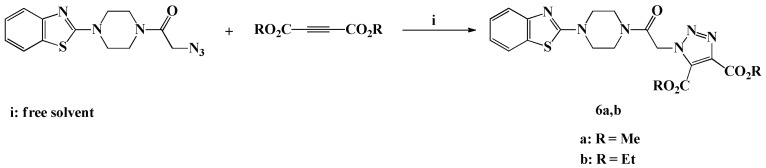
Solvent free click synthesis of dimethyl/diethyl 1-(2-(4-(benzothiazol-2-yl)piperazin-1-yl)-2-oxoethyl)-1*H*-1,2,3-triazole-4,5-dicarboxylate (**6a**,**b**).

The synthetic protocols used for the synthesis of the desired bioactive compounds have been depicted in Scheme 1, Scheme 2, Scheme 3 and Scheme 4. The precursor 2-azido-1-(4-(benzo[*d*]thiazol-2-yl)piperazin-1-yl)ethanone (**3**) required for the 1,3-dipolar cycloaddition reaction has been synthesized via, first base assisted acylation of 2-(piperazin-1-yl)benzo[*d*]thiazole (**1**) with bromoacetyl bromide, in the presence of triethylamine in dichloromethane at room temperature, to afford the bromoacetylpiperazine intermediate **2,** which upon treatment with sodium azide in a mixture of acetone-water (4:1), furnished the targeted azide **3** in 91% yield (Scheme 1).

The 2-(1-piperazinyl)benzothiazole [33] was prepared from 2-chlorobenzothiazole by nucleophilic substitution of the chlorine atom by piperazine in the presence of sodium bicarbonate in an aqueous solution of 2-propanol, followed by purification with column chromatography.

The structure of the newly synthesized compound **2** is in agreement with IR, ^1^H-NMR, ^13^C-NMR and mass spectra. Its IR spectrum showed clearly the absence of the piperazine amino group and the presence of a strong absorption band at 1685 cm^−1^ attributed to the acetyl carbonyl (C=O) group. Moreover, the ^1^H-NMR confirmed the absence of NH proton, which was clear evidence of the success of the acylation of the piperazine nucleus. The spectrum exhibited characteristic singlet at δ_H_ 3.33 ppm assigned to the methylene acetyl protons (CH_2_CO). The aromatic and aliphatic protons resonated at their expected chemical shifts. Structural assignment of the acetyl side chain in the structure of compound **2** has been also supported by ^13^C-NMR analysis through the appearance of two characteristic signals at δ_C_ 59.8 and δ_C_ 169.7 ppm belonging to the CH_2_ and C=O carbons, respectively. The formation of the azido derivative **3** was also established on the basis of its spectroscopic data. In the IR spectrum, the presence of the ki azido group in its structure was confirmed by the appearance of an absorption band at 2170 cm^−1^. In contrast, no change has been observed in the protons and carbons assignment to the azido derivative **3** compared to its corresponding precursor **2,** except a downfield shift of the CH_2_CO protons from δ_H_ 3.33 ppm for compound **2** to δ_H_ 4.22 ppm for compound **3** (See experimental section).

In the presence of copper sulfate and sodium ascorbate as catalysts and DMSO/H_2_O (1:1) as the solvent, 1,3-dipolar cycloaddition reaction of the synthesized azide **3** with commercially available functionalized alkynes **4a**–**e** resulted on the formation of novel benzothiazole-piperazine-1,2,3-triazole hybrids **5a**–**e** carrying different hydroxylated and/or ester based alkyl side chains (Scheme 2). The click synthesis required stirring at 80 °C for 8 h to afford the desired 1,2,3-triazole hybrids **5a**–**e** in 80–90% yield. The copper catalyst was implemented to ensure the regioselective formation of the 1,4-disubstituted 1,2,3-triazole isomers **5a**–**e**.

The success of the click synthesis of the 1,4-disubstituted-1,2,3-triazole hybrids **5a**–**e** has been clearly evidenced by ^1^H-NMR analysis through the appearance of one distinct singlet in the aromatic region at δ_H_ 7.76–8.66 ppm attributable the 1,2,3-triazolyl proton. The OH proton of the hydroxylated alkyl residue linked to the 1,2,3-triazole ring resonated as a triplet at δ_H_ 5.22 ppm for compound **5a** and as a broad singlet at δ_H_ 4.44–6.02 ppm for compounds **5b**–**d**. Moreover, the ester functionality characterizing the 1,2,3-triazole **5e** appeared as a triplet at δ_H_ 1.32 ppm and a quartet at δ_H_ 4.30–4.35 ppm assigned to the ester methyl and methylene protons, respectively. In the ^13^C-NMR spectra, the absence of signals on the Sp-carbon regions and appearance of new signals in the aliphatic area assigned to the alkyl side chain carbons confirmed the success of the cycloaddition reaction.

Under the same optimized copper (I) catalyzed click synthesis, a new library of benzothiazole-piperazine-1,2,3-triazole hybrids **5f**–**l** tagged different heterocyclic scaffolds, including 1,2,4-triazole, benzothiazole, benzimidazole and/or isatin, has been successfully designed and synthesized in 87–90% yield through the ligation of compound **3** with the appropriate heterocyclic alkynes **4f**–**l**. It should be noted that the propargylated heterocyclic precursors **4f**–**l** needed for the construction of compounds **5f**–**l** were synthesized via the alkylation of 4,5-disubstituted-1,2,4-triazole-3-thiones, 2-mercaptobenzothiazole, 2-mercaptobenzimidazole and/or isatin with propargyl bromide, in the presence of potassium carbonate in DMF at room temperature. The ^1^H-NMR spectra of compounds **5f**–**l** were fully characterized by the appearance of two new diagnostic singlets at δ_H_ 4.44–5.00 and δ_H_ 7.90–8.09 ppm assigned to the methylene SCH_2_/NCH_2_ protons and the triazolic H-5 proton, respectively. The spectra also revealed the presence of 4–10 extra aromatic protons in the aromatic region related to the aromatic heterocyclic alkyne building blocks, which confirm their incorporation through 1,3-dipolar cycloaddition. The ^13^C-NMR analysis also confirmed the success of the click synthesis through; first the absence of the Sp-carbon signals from their usual chemical shift region, and second the presence of new signal in the upfield region at 25.5–35.5 ppm assignable to the SCH_2_/NCH_2_ carbon.

As continuation of our previous study directed to design novel 1,4,5-trisubstituted-1,2,3-triazoles [34,35,36], we have adopted the optimized ecofriendly solvent free click procedure previously developed in our laboratory for the elaboration of two novel 4,5-diester-1,2,3-triazoles carrying benzothiazole-piperazine conjugate. The reaction required 1,3-dipolar cycloaddition reaction between the azide **3** with dimethyl/diethylacetylene dicarboxylate under neat conditions, in a water bath for 3 min, to afford the desired dimethyl/diethyl 1-(2-(4-(benzothiazol-2-yl)piperazin-1-yl)-2-oxoethyl)-1*H*-1,2,3-triazole-4,5-dicarboxylate (**6a**,**b**) in 92–94% yields.

The newly synthesized 4,5-disubstituted 1,2,3-triazoles **6a**,**b** are in agreement with the IR, ^1^H-NMR, ^13^C-NMR and HRMS spectral data. In the ^1^H-NMR spectrum of compound **6a**, the two non-equivalents methyl ester protons were recorded as two characteristic singlets at δ_H_ 3.97 and δ_H_ 4.00 ppm. The ethyl ester protons attributable to compound **6b** also appeared as two sets of multiplets at δ_H_ 1.24–1.33 and δ_H_ 4.29–4.39 ppm, respectively. The methoxy and ethoxy carbons characteristic of the two non-equivalent ester functionalities resonated at their expected chemical shift in the aliphatic region at δ_C_ 14.1–14.4 and δ_C_ 52.7–62.8 ppm. In addition, the carbonyl ester carbons appeared in the downfield region at δ_C_ 168.2–168.5 ppm.

### 2.2. Antiproliferative Activity

Compounds **2**, **3**, **5a**–**l,** and **6a**,**b** were tested for their in vitro antiproliferation activities against two human breast cancer cell lines; MCF7 and T47D, and two human colon cancer cell lines; HCT116 and Caco2 according to the protocol described in the ISO 10993-5 guide [37]. As shown in Table 1, relative to the parent compounds **2** and **3**, some of the hybrid compounds exhibited good antiproliferative activity against both breast and colon carcinoma cell lines. Among the synthesized compounds, the 3-hydroxypropyl hybrid compound **5b** has demonstrated the most potent antiproliferative activity with IC_50_ of 38 µM and 33 µM against the breast carcinoma cell lines of T47D and MCF7, respectively, and IC_50_ of 48 µM and 42 µM against the colon carcinoma cell lines of HCT116 and Caco2, respectively.

In addition, title compounds **5c**, **5d**, **5h,** and **5g** tethering 1,4-disubstituted-1,2,3-triazoles have exhibited appreciable activity against all studied cell lines. From the trend activity, cell proliferation assay demonstrates that hybrid molecules tethering ethyl ester substitution on the triazole moiety is less favored for anticancer activity (title compound **5e**). On the other hand, aryl and alkyl substituted triazole is favored for anticancer activity. The thiomethyl-triazole substituent exhibited variable effects on the anticancer activity profile that appeared to be dependent on the substituent’s size and position on the terminal triazole ring. For example, the 5-methyl-4-phenyl thiomethyl-triazole substitution (title compound **5f**) had atrocious effect on activity, while the 4-methyl-5-phenyl thiomethyl-triazole substitution (title compound **5g**) exhibited more potent antiproliferative activity against all examined cancer cell lines. Interestingly, the hybrid molecules with 1,2,3-triazole tri-substitutions (title compounds **6a** and **6b**) have shown poor antiproliferative profile against all cancer cell lines in this study.

### 2.3. In Silico ADME and LogP Analysis

In silico ADME (adsorption, distribution, metabolism, and excretion) was performed to confirm the reliability in vitro biological activity. Evaluation of in silico ADME is a reliable technique to confirm the potential of a drug candidate. Before going in clinical trial, preliminary agreement is needed for ADME and can be easily provided [38]. For good absorption and permeability, cLog*P* should be less than 5. The hydrophilicity and cLog*P* values are correlated because hydrophilicity depends on, and is expressed in terms of, the cLog*P* value. Any drug to be active should not have more than one violation [39]. To qualify the preliminary requirement, log*P* and ADME analysis have preformed for synthesized benzothiazole-piperazine conjugates (**2**, **3**, **5a**–**5l**, and **6a**,**b**).

Violations of Lipinski’s rule and predicted ADME parameters (molecular weight (MW), log*P*, topological polar surface are (tPSA), number of hydrogen donors (nON), and acceptors (nOHNH) and volume) are presented in Table 2. From Table 2, screening data for ADME and log*P* revealed that all compounds are safe. According to these data, compounds comply Lipinski’s rule of five and number of violation except compound **5i**. The ADME parameters are in good agreement and may have good pharmacokinetic profile with good lipophilicity.

## 3. Materials and Methods

### 3.1. General

All melting points were measured on a Stuart Scientific SMP1 apparatus (Red Hill, UK) and are uncorrected. The IR spectra were measured in a KBr matrix with a Perkin-Elmer 1430 series FTIR spectrometer (PerkinElmer, Santa Clara, California, USA). The NMR spectra were recorded with an Advance Bruker spectrophometer (Fällanden, Switzerland) at 400 MHz for the ^1^H-NMR analysis and at 100 MHz for the ^13^C-NMR analysis, using Tetramethylsilane (TMS) (0.00 ppm) as the internal. A Finnigan MAT 95XL spectrometer (Darmstadt, Germany) was used for the determination of the EI mass spectra. Elemental analyses were performed using a GmbH-Vario EL III Element Analyzer (Hanau, Germany). In silicon ADME and log*P* value has been calculated using Molinspiration Cheminformatics software (Nova ulica 61, SK-900 26 Slovensky Grob, Slovak Republic) on http://www.molinspiration.com.

#### 3.1.1. Synthesis and Characterization of 1-(4-(Benzo[*d*]thiazol-2-yl)piperazin-1-yl)-2-bromoethanone (**2**)

Bromoacetyl bromide (10 mmol) was added dropwise to a mixture of 2-(piperazin-1-yl)benzothiazole (**1**) (10 mmol) and triethylamine (10 mmol) in dichloromethane (30 mL) at 0 °C with stirring. The stirring was continued at room temperature for 6 h. The solid thus formed was filtered, washed with water and recrystallized from ethanol.

White pellets, 89%, m.p. 88–90 °C. IR (*υ*, cm^−1^): 1570 (C=C), 1615 (C=N), 1685 (C=O), 2920 (C-H al), 3045 (C-H ar). ^1^H-NMR: δ 2.67 (t, 4H, *J* = 4 Hz, 2 × NCH_2_), 3.33 (s, 2H, CH_2_Br), 3.57 (t, 4H, *J* = 4 Hz, 2 × NCH_2_), 7.05–7.09 (m, 1H, Ar-H), 7.26–7.30 (m, 1H, Ar-H), 7.47 (d, 1H, *J* = 8 Hz, Ar-H), 7.77 (d, 1H, *J* = 8 Hz, Ar-H). ^13^C-NMR: δ 47.9, 51.0, 58.0, 59.8 (CH_2_); 118.5, 121.1, 121.2, 125.9, 130.3, 152.4, 168.0, 169.7 (Ar-C, C=N, C=O) ppm. EI-MS (*m*/*z*): 339.00 (M^+^). Anal. Calcd for C_13_H_14_BrN_3_OS: C 45.89; and H 4.15 N 12.35. Found: C 45.77; H 4.22; N 12.26.

#### 3.1.2. Synthesis and Characterization of 2-Azido-1-(4-(benzo[*d*]thiazol-2-yl)piperazin-1-yl)ethanone (**3**)

A mixture of compound **2** (10 mmol) and sodium azide (12 mmol) in a mixture of acetone:water (4:1) (100 mL) was stirred for 24 h at room temperature. The excess of solvent was evaporated under vacuum. Compound **3** was collected by filtration, washed with water and recrystallized from ethanol.

White pellets; 91%; m.p. 148–150 °C. IR (*υ*, cm^−1^): 1560 (C=C), 1620 (C=N), 1690 (C=O), 2170 (N=N=N), 2930 (C-H al), 3070 (C-H ar). ^1^H-NMR: δ 3.49–3.66 (m, 8H, 4 × NCH_2_), 4.22 (s, 2H, CH_2_N_3_), 7.07–7.11 (m, 1H, Ar-H), 7.27–7.31 (m, 1H, Ar-H), 7.49 (d, 1H, *J* = 8 Hz, Ar-H), 7.80 (d, 1H, *J* = 8 Hz, Ar-H). ^13^C-NMR: δ 40.7, 43.3, 47.6, 49.7 (CH_2_); 118.7, 121.2, 121.4, 126.0, 130.3, 152.2, 166.1, 167.9 (Ar-C, C=N, C=O) ppm. EI-MS (*m*/*z*): 302.10 (M^+^). Anal. Calcd for C_13_H_14_N_6_OS: C 51.64; H 4.67; and N 27.80. Found: C 51.73; H 4.60; N 27.74.

#### 3.1.3. General Procedure for the Synthesis of Propargylated Heterocycles **4f**–**l**

Potassium carbonate (11 mmol) was added to a stirred solution of compound **1** (10 mmol) dissolved in DMF (25 mL) and stirring was continued for 2 h. Then, propargyl bromide (11 mmol) was added and the reaction mixture was stirred at room temperature overnight. The mixture was poured onto crushed ice water and the precipitate thus formed was collected by filtration and recrystallized from ethanol to afford the desired alkynes **4f**–**l**.

#### 3.1.4. Characterization of 5-Methyl-4-phenyl-3-(prop-2-yn-1-ylthio)-1,2,4-triazole (**4f**)

Colorless crystals, 93%, m.p. 103–104 °C. IR (*υ*, cm^−1^): 1560 (C=C), 1610 (C=N), 2140 (C≡C), 2945 (C-H al), 3040 (C-H ar), 3330 (≡CH). (400 MHz, CDCl_3_): δ 2.24 (s, 1H, ≡CH), 2.32 (s, 3H, CH_3_), 3.94 (s, 2H, SCH_2_), 7.25–7.28 (m, 3H, Ar-H), 7.55–7.57 (m, 2H, Ar-H). ^13^C-NMR: δ 11.3 (CH_3_); 21.2 (SCH_2_); 72.3, 78.2 (C≡C); and 126.9, 130.0, 130.1, 133.2, 149.5, 153.1 (Ar-C, C=N) ppm.

#### 3.1.5. Characterization of 4-Methyl-5-phenyl-3-(prop-2-yn-1-ylthio)-1,2,4-triazole (**4g**)

Colorless crystals, 91%, m.p. 115–116 °C. IR (*υ*, cm^−1^): 1575 (C=C), 1610 (C=N), 2150 (C≡C), 2960 (C-H al), 3050 (C-H ar), 3300 (≡CH). ^1^H-NMR: δ 3.22 (s, 1H, ≡CH), 3.39 (s, 3H, CH_3_), 4.40 (s, 2H, SCH_2_), 7.28–7.33 (m, 3H, Ar-H), 7.65–7.70 (m, 2H, Ar-H). ^13^C-NMR: δ 27.7 (SCH_2_); 37.6 (CH_3_); 73.4, 78.2 (C≡C); and 126.2, 129.9, 130.5, 130.8, 134.8, 149.9, 154.8 (Ar-C, C=N) ppm.

#### 3.1.6. Characterization of 4-Ethyl-5-phenyl-3-(prop-2-yn-1-ylthio)-1,2,4-triazole (**4h**)

Colorless crystals, 93%, m.p. 108–109 °C. IR (*υ*, cm^−1^): 1575 (C=C), 1615 (C=N), 2155 (C≡C), 2935 (C-H al), 3050 (C-H ar), 3320 (≡CH). ^1^H-NMR: δ 1.15 (s, 3H, *J* = 8 Hz, CH_3_), 3.25 (s, 1H, ≡CH), 3.82–3.89 (q, 2H, NCH_2_CH_3_), 4.45 (s, 2H, SCH_2_), 7.26–7.30 (m, 3H, Ar-H), 7.60–7.66 (m, 2H, Ar-H). ^13^C-NMR: δ 15.8 (CH_3_); 28.4 (SCH_2_); 39.3 (NCH_2_CH_3_); 74.0, 79.4 (C≡C); and 126.1, 129.6, 130.1, 130.4, 134.6, 150.1, 155.3 (Ar-C, C=N) ppm.

#### 3.1.7. Characterization of 4-Phenyl-5-phenyl-3-(prop-2-yn-1-ylthio)-1,2,4-triazole (**4i**)

Colorless crystals, 92%, m.p. 103–104 °C. IR (*υ*, cm^−1^): 1580 (C=C), 1605 (C=N), 2145 (C≡C), 2920 (C-H al), 3035 (C-H ar), 3310 (≡CH). ^1^H-NMR: δ 3.28 (s, 1H, ≡CH), 4.00 (s, 2H, SCH_2_), 7.35–7.43 (m, 7H, Ar-H), 7.54–7.56 (m, 3H, Ar-H). ^13^C-NMR: δ 20.9 (SCH_2_); 74.7, 79.4 (C≡C); and 126.5, 127.7, 128.0, 128.6, 129.8, 130.0, 130.1, 133.7, 150.4, 154.6 (Ar-C, C=N) ppm.

#### 3.1.8. Characterization of 2-(Prop-2-yn-1-ylthio)benzo[d]thiazole (**4j**)

White pellets, 92%, m.p. 82–83 °C. IR (*υ*, cm^−1^): 1590 (C=C), 1620 (C=N), 2140 (C≡C), 2955 (C-H al), 3050 (C-H ar), 3300 (≡CH). (400 MHz, CDCl_3_): δ 2.33 (s, 1H, ≡CH), 4.16 (s, 2H, SCH_2_), 7.33 (t, 1H, *J* = 8 Hz, Ar-H), 7.45 (t, 1H, *J* = 8 Hz, Ar-H), 7.79 (d, 1H, *J* = 8 Hz, Ar-H), 7.94 (d, 1H, *J* = 8 Hz, Ar-H). ^13^C-NMR: δ 21.6 (SCH_2_); 72.3, 78.3 (C≡C); and 121.1, 121.8, 124.5, 126.2, 135.4, 142.5, 153.0, 164.6 (Ar-C, C=N) ppm.

#### 3.1.9. Characterization of 2-(Prop-2-yn-1-ylthio)benzo[d]imidazole (**4k**)

Colorless crystals, 89%, m.p. 149–150 °C. IR (*υ*, cm^−1^): 1580 (C=C), 1615 (C=N), 2155 (C≡C), 2960 (C-H al), 3060 (C-H ar), 3280–3340 (N-H, ≡CH). ^1^H-NMR: δ 3.20 (s, 1H, ≡CH), 4.16 (s, 2H, SCH_2_), 7.14–7.16 (m, 2H, Ar-H), 7.46–7.51 (m, 2H, Ar-H), 12.65 (s, 1H, NH). ^13^C-NMR: δ 20.6 (SCH_2_); 74.5, 80.5 (C≡C); and 110.9, 118.0, 122.1, 135.9, 144.1, 148.8 (Ar-C, C=N) ppm.

#### 3.1.10. Characterization of 1-(Prop-2-yn-1-yl)indoline-2,3-dione (**5l**)

Orange crystals, 94%, m.p. 157–158 °C. IR (*υ*, cm^−1^): 1580 (C=C), 1615 (C=N), 1710 (C=O), 2150 (C≡C), 2950 (C-H al), 3060 (C-H ar), 3315 (≡CH). (400 MHz, CDCl_3_): δ 2.33 (s, 1H, ≡CH), 7.14–7.21 (m, 2H, Ar-H), 7.64–7.68 (m, 2H, Ar-H). ^13^C-NMR: δ 29.4 (SCH_2_); 73.3, 75.6 (C≡C); and 111.1, 117.6, 124.2, 125.4, 138.5, 149.6, 151.1, 182.5 (Ar-C, C=N, C=O) ppm.

#### 3.1.11. General Procedure for the Synthesis of 1,4-Disubstituted 1,2,3-triazoles (**5a**–**l**)

A solution of copper sulfate (0.8 mmol) and sodium ascorbate (1.1 mmol) in water (10 mL) was added with stirring to a mixture of the appropriate alkyne **4a**–**l** (1 mmol) and benzothiazoleazide **3** (1 mmol) in DMSO (10 mL). Then, the reaction mixture was heated at 80 °C for 8 h, until the consumption of the starting material as indicated by TLC. The reaction mixture was quenched with ice water and the solid thus formed was collected by filtration, washed with saturated solution of ammonium chloride and recrystallized from ethanol to give the desired 1,2,3-triazoles **5a**–**l**.

#### 3.1.12. Characterization of 1-(4-(Benzothiazol-2-yl)piperazin-1-yl)-2-(4-(hydroxymethyl)-1*H*-1,2,3-triazol-1-yl)ethanone (**5a**)

Colorless needles, 86%, m.p. 205-206 °C. IR (*υ*, cm^−1^): 1570 (C=C), 1615 (C=N), 1685 (C=O), 2965 (C-H al), 3050 (C-H ar), 3310 (O-H). ^1^H-NMR: δ 3.62-3.71 (m, 8H, 4 × NCH_2_), 4.55 (d, 2H, *J* = 4 Hz, OCH_2_), 5.22 (t, 1H, *J* = 4 Hz, OH), 5.53 (s, 2H, CH_2_CO), 7.11 (t, 1H, *J* = 8 Hz, Ar-H), 7.31 (t, 1H, *J* = 8 Hz, Ar-H), 7.51 (d, 1H, *J* = 8 Hz, Ar-H), 7.82 (d, 1H, *J* = 8 Hz, Ar-H), 7.87 (s, 1H, CH-1,2,3-triazole). ^13^C-NMR: δ 41.3, 44.0, 48.0, 48.2, 51.0 (CH_2_); 55.5 (OCH_2_); 119.2, 121.7, 121.9, 124.8, 126.5, 130.9, 148.2, 152.7, 165.1, 168.5 (Ar-C, C=N, C=O) ppm. EI-MS (*m*/*z*): 358.12 (M^+^). Anal. Calcd for C_16_H_18_N_6_O_2_S: C 53.62; H 5.06; N 23.45. Found: C 53.73; H 5.11; N 23.38.

#### 3.1.13. Characterization of 1-(4-(Benzothiazol-2-yl)piperazin-1-yl)-2-(4-(3-hydroxypropyl)-1*H*-1,2,3-triazol-1-yl)ethanone (**5b**)

White solid, 84%, m.p. 229–230 °C. IR (*υ*, cm^−1^): 1560 (C=C), 1610 (C=N), 1700 (C=O), 2980 (C-H al), 3020 (C-H ar), 3310 (O-H). ^1^H-NMR: δ1.74–1.77 (m, 2H, CH_2_CH_2_CH_2_), 2.67 (t, 2H, *J* = 8 Hz, CH_2_CH_2_), 3.45 (t, 2H, *J* = 8 Hz, CH_2_O), 3.62–3.70 (m, 8H, 4 × NCH_2_), 4.44 (bs, 1H, OH), 5.48 (s, 2H, CH_2_CO), 7.10 (t, 1H, *J* = 8 Hz, Ar-H), 7.30 (t, 1H, *J* = 8 Hz, Ar-H), 7.51 (d, 1H, *J* = 8 Hz, Ar-H), 7.76 (s, 1H, CH-1,2,3-triazole), 7.81 (d, 1H, *J* = 8 Hz, Ar-H). ^13^C-NMR: δ 21.6 (CH_2_CH_2_CH_2_), 32.2 (CH_2_CH_2_), 40.8, 43.5, 47.5, 50.5 (CH_2_); 60.0 (OCH_2_); 117.1, 119.6, 119.8, 124.4, 128.8, 150.6, 163.0, 166.4 (Ar-C, C=N, C=O) ppm. EI-MS (*m*/*z*): 386.15 (M^+^). Anal. Calcd for C_18_H_22_N_6_O_2_S: C, 55.94; H, 5.74; N, 21.75. Found: C 55.75; H 5.69; N 21.87.

#### 3.1.14. Characterization of 1-(4-(Benzothiazol-2-yl)piperazin-1-yl)-2-(4-(hydroxy(phenyl)methyl)-1*H*-1,2,3-triazol-1-yl)ethanone (**5c**)

Brown solid, 82%, m.p. 249–250 °C. IR (*υ*, cm^−1^): 1550 (C=C), 1610 (C=N), 1690 (C=O), 2940 (C-H al), 3030 (C-H ar), 3285 (O-H). ^1^H-NMR: δ 3.61–3.69 (m, 8H, 4 × NCH_2_), 5.49 (s, 2H, CH_2_CO), 5.84 (s, 1H, CH), 6.02 (bs, 1H, OH), 7.10 (t, 1H, *J* = 8 Hz, Ar-H), 7.31–7.36 (m, 4H, Ar-H), 7.42–7.51 (m, 3H, Ar-H), 7.77–7.81 (m, 2H, Ar-H and CH-1,2,3-triazole). ^13^C-NMR: δ 41.3, 44.0, 48.0, 48.1, 51.0 (CH_2_); 68.4 (CH); 119.2, 121.7, 121.9, 124.8, 126.5, 126.8, 127.5, 128.5, 130.3, 130.9, 144.6, 152.7, 165.0, 168.5 (Ar-C, C=N, C=O) ppm. EI-MS (*m*/*z*): 434.15 (M^+^). Anal. Calcd for C_22_H_22_N_6_O_2_S: C, 60.81; H, 5.10; N, 19.34. Found: C 60.73; H 5.16; N 19.42.

#### 3.1.15. Characterization of 1-(4-(Benzothiazol-2-yl)piperazin-1-yl)-2-(4-(hydroxydiphenylmethyl)-1*H*-1,2,3-triazol-1-yl)ethanone (**5d**)

Brown solid, 80%, m.p. 239–240 °C. IR (*υ*, cm^−1^): 1565 (C=C), 1620 (C=N), 1615 (C=O), 2950 (C-H al), 3075 (C-H ar), 3335 (O-H). ^1^H-NMR: δ 3.60–3.68 (m, 8H, 4 × NCH_2_), 5.46 (s, 2H, CH_2_CO), 5.92 (bs, 1H, OH), 7.07–7.15 (m, 3H, Ar-H), 7.28–7.34 (m, 6H, Ar-H), 7.40–7.50 (m, 4H, Ar-H), 7.75–7.85 (m, 2H, Ar-H and CH-1,2,3-triazole). ^13^C-NMR: δ 41.5, 44.1, 48.1, 48.2, 51.2 (CH_2_); 119.1, 120.2, 121.6, 121.8, 124.6, 124.9, 126.3, 126.5 126.8, 127.4, 128.6, 130.2, 130.8, 144.7, 152.8, 165.1, 168.3 (Ar-C, C=N, C=O) ppm. EI-MS (*m*/*z*): 510.18 (M^+^). Anal. Calcd for C_28_H_26_N_6_O_2_S: C 65.86; H 5.13; N 16.46. Found: C 65.71; H 5.19; N 16.49.

#### 3.1.16. Characterization of Ethyl 1-(2-(4-(Benzothiazol-2-yl)piperazin-1-yl)-2-oxoethyl)-1*H*-1,2,3-triazole-4-carboxylate (**5e**)

Colorless needles, 90%, m.p. 200–202 °C [decomposition]. IR (*υ*, cm^−1^): 1575 (C=C), 1610 (C=N), 1710 (C=O), 2940 (C-H al), 3035 (C-H ar). ^1^H-NMR: δ 1.32 (t, 3H, *J* = 8 Hz, CH_3_), 3.63–3.73 (m, 8H, 4 × NCH_2_), 4.30–4.35 (q, 2H, OCH_2_), 5.65 (s, 2H, CH_2_CO), 7.11 (t, 1H, *J* = 8 Hz, Ar-H), 7.31 (t, 1H, *J* = 8 Hz, Ar-H), 7.52 (d, 1H, *J* = 8 Hz, Ar-H), 7.82 (d, 1H, *J* = 8 Hz, Ar-H), 8.66 (s, 1H, CH-1,2,3-triazole). ^13^C-NMR: δ 14.6 (CH_3_); 41.5, 44.0, 48.0, 48.1, 51.5 (CH_2_); 61.0 (OCH_2_); 119.2, 121.7, 121.9, 126.5, 130.9, 131.3, 139.1, 152.7, 160.7, 164.6, 168.5 (Ar-C, C=N, C=O) ppm. EI-MS (*m*/*z*): 400.13 (M^+^). Anal. Calcd for C_18_H_20_N_6_O_3_S: C 53.99; H 5.03; N 20.99. Found: C 53.84; H 5.10; N 20.87.

#### 3.1.17. Characterization of 1-(4-(Benzo[*d*]thiazol-2-yl)piperazin-1-yl)-2-(4-(((5-methyl-4-phenyl-4*H*-1,2,4-triazol-3-yl)thio)methyl)-1*H*-1,2,3-triazol-1-yl)ethanone (**5f**)

White pellets, 88%, m.p. 130–132 °C. IR (*υ*, cm^−1^): 1565 (C=C), 1620 (C=N), 1680 (C=O), 2915(C-H al), 3020 (C-H ar). ^1^H-NMR: δ 2.21 (s, 3H, CH_3_), 3.62–3.70 (m, 8H, 4 × NCH_2_), 4.44 (s, 2H, SCH_2_), 5.54 (s, 2H, CH_2_CO), 7.10 (t, 1H, *J* = 8 Hz, Ar-H), 7.31–7.40 (m, 3H, Ar-H), 7.49–7.58 (m, 4H, Ar-H), 7.82 (d, 1H, *J* = 8 Hz, Ar-H), 7.94 (s, 1H, CH-1,2,3-triazole). ^13^C-NMR: δ 11.5 (CH_3_); 27.6 (SCH_2_); 41.4, 44.0, 48.0, 48.1, 51.3 (CH_2_); 119.2, 121.7, 121.9, 124.8, 126.5, 127.6, 130.3, 130.4, 130.8, 133.6, 152.7, 164.9, 168.5 (Ar-C, C=N, C=O) ppm. EI-MS (*m*/*z*): 531.16 (M^+^). Anal. Calcd for C_25_H_25_N_9_OS_2_: C 56.48; H 4.74; N 23.71. Found: C 56.63; H 4.69; N 23.82.

#### 3.1.18. Characterization of 1-(4-(Benzo[*d*]thiazol-2-yl)piperazin-1-yl)-2-(4-(((4-methyl-5-phenyl-4*H*-1,2,4-triazol-3-yl)thio)methyl)-1*H*-1,2,3-triazol-1-yl)ethanone (**5g**)

White pellets, 87%, m.p. 196–198 °C. IR (*υ*, cm^−1^): 1570 (C=C), 1615 (C=N), 1680 (C=O), 2940 (C-H al), 3035 (C-H ar). ^1^H-NMR: δ 3.36 (s, 3H, CH_3_), 3.51-3.69 (m, 8H, 4 × NCH_2_), 4.47 (s, 2H, SCH_2_), 5.55 (s, 2H, CH_2_CO), 7.10 (t, 1H, *J* = 8 Hz, Ar-H), 7.29 (t, 1H, *J* = 8 Hz, Ar-H), 7.49–7.57 (m, 4H, Ar-H), 7.73–7.81 (m, 3H, Ar-H), 7.96 (s, 1H, CH-1,2,3-triazole). ^13^C-NMR: δ 27.9 (SCH_2_); 32.4 (CH_3_); 41.4, 44.0, 48.0, 48.1, 51.3 (CH_2_); 119.2, 121.7, 121.9, 124.8, 126.5, 128.8, 129.3, 130.4, 130.9, 152.7, 164.9, 168.5 (Ar-C, C=N, C=O) ppm. EI-MS (*m*/*z*): 531.16 (M^+^). Anal. Calcd for C_25_H_25_N_9_OS_2_: C 56.48; H 4.74; N 23.71. Found: C 56.55; H 4.80; N 23.79.

#### 3.1.19. Characterization of 1-(4-(Benzo[*d*]thiazol-2-yl)piperazin-1-yl)-2-(4-(((4-ethyl-5-phenyl-4*H*-1,2,4-triazol-3-yl)thio)methyl)-1*H*-1,2,3-triazol-1-yl)ethanone (**5h**)

White pellets, 89%, m.p. 217–219 °C. IR (*υ*, cm^−1^): 1565 (C=C), 1615 (C=N), 1695 (C=O), 2945 (C-H al), 3040 (C-H ar). ^1^H-NMR: δ 1.11 (s, 3H, *J* = 8 Hz, CH_3_), 3.62–3.69 (m, 8H, 4 × NCH_2_), 3.88–3.92 (q, 2H, NCH_2_CH_3_), 4.52 (s, 2H, SCH_2_), 5.54 (s, 2H, CH_2_CO), 7.11 (t, 1H, *J* = 8 Hz, Ar-H), 7.31 (t, 1H, *J* = 8 Hz, Ar-H), 7.49–7.68 (m, 6H, Ar-H), 7.82 (d, 1H, *J* = 8 Hz, Ar-H), 7.90 (s, 1H, CH-1,2,3-triazole). ^13^C-NMR: δ 15.4 (CH_3_); 28.6 (SCH_2_); 39.7 (NCH_2_CH_3_); 41.4, 44.0, 48.0, 48.1, 51.2 (CH_2_); 119.2, 121.7, 121.9, 125.8, 126.5, 127.8, 128.9, 129.4, 130.5, 130.9, 152.7, 165.0, 168.5 (Ar-C, C=N, C=O) ppm. EI-MS (*m*/*z*): 545.18 (M^+^). Anal. Calcd for C_26_H_27_N_9_OS_2_: C 57.23; H 4.94; N 23.10. Found: C 57.09; H 4.88; N 23.20.

#### 3.1.20. Characterization of 1-(4-(Benzo[*d*]thiazol-2-yl)piperazin-1-yl)-2-(4-(((4,5-diphenyl-4*H*-1,2,4-triazol-3-yl)thio)methyl)-1*H*-1,2,3-triazol-1-yl)ethanone (**5i**)

White pellets, 90%, m.p. 276–278 °C [dec]. IR (*υ*, cm^−1^): 1570 (C=C), 1610 (C=N), 1690 (C=O), 2940 (C-H al), 3045 (C-H ar). ^1^H-NMR: δ 3.61–3.69 (m, 8H, 4 × NCH_2_), 4.50 (s, 2H, SCH_2_), 5.53 (s, 2H, CH_2_CO), 7.11 (t, 1H, *J* = 8 Hz, Ar-H), 7.31–7.36 (m, 8H, Ar-H), 7.49–7.54 (m, 4H, Ar-H), 7.82 (d, 1H, *J* = 8 Hz, Ar-H), 7.95 (s, 1H, CH-1,2,3-triazole). ^13^C-NMR: δ 27.5 (SCH_2_); 41.4, 44.0, 48.0, 48.1, 51.2 (CH_2_); 119.2, 121.7, 121.9, 125.9, 126.5, 127.0, 128.1, 128.4, 129.0, 130.2, 130.3, 130.5, 130.9, 134.2, 142.6, 151.7, 152.7, 154.9, 165.0, 168.5 (Ar-C, C=N, C=O) ppm. EI-MS (*m*/*z*): 593.17 (M^+^). Anal. Calcd for C_30_H_27_N_9_OS_2_: C 60.69; H 4.58; N 21.23. Found: C 60.77; H 4.63; N 21.29.

#### 3.1.21. Characterization of 1-(4-(Benzo[*d*]thiazol-2-yl)piperazin-1-yl)-2-(4-((benzo[*d*]thiazol-2-ylthio)methyl)-1*H*-1,2,3-triazol-1-yl)ethanone (**5j**)

White pellets, 87%, m.p. 228–229 °C [decomposition]. IR (*υ*, cm^−1^): 1580 (C=C), 1625 (C=N), 1685 (C=O), 2965 (C-H al), 3040 (C-H ar). ^1^H-NMR: δ 3.60–3.68 (m, 8H, 4 × NCH_2_), 4.74 (s, 2H, SCH_2_), 5.53 (s, 2H, CH_2_CO), 7.10 (t, 1H, *J* = 8 Hz, Ar-H), 7.30 (t, 1H, *J* = 8 Hz, Ar-H), 7.38 (t, 1H, *J* = 8 Hz, Ar-H), 7.48–7.52 (m, 2H, Ar-H), 7.59 (d, 1H, *J* = 8 Hz, Ar-H), 7.81 (d, 1H, *J* = 8 Hz, Ar-H), 7.92 (d, 1H, *J* = 8 Hz, Ar-H), 8.02–8.04 (m, 2H, Ar-H and CH-1,2,3-triazole). ^13^C-NMR: δ 27.8 (SCH_2_); 41.3, 44.0, 48.0, 48.1, 51.2 (CH_2_); 119.2, 121.7, 121.9, 122.3, 125.0, 126.0, 126.5, 126.8, 130.8, 135.1, 142.5, 152.7, 153.0, 164.9, 166.3, 168.5 (Ar-C, C=N, C=O) ppm. EI-MS (*m*/*z*): 507.10 (M^+^). Anal. Calcd for C_23_H_21_N_7_OS_3_: C 54.42; H 4.17; N 19.31. Found: C 54.31; H 4.24; N 19.39.

#### 3.1.22. Characterization of 2-(4-(((1*H*-Benzo[*d*]imidazol-2-yl)thio)methyl)-1*H*-1,2,3-triazol-1-yl)-1-(4-(benzo[*d*]thiazol-2-yl)piperazin-1-yl)ethanone (**5k**)

White pellets, 85%, m.p. 182–184 °C. IR (*υ*, cm^−1^): 1575 (C=C), 1620 (C=N), 1690 (C=O), 2970 (C-H al), 3070 (C-H ar), 3280 (N-H). ^1^H-NMR: δ 3.63–3.70 (m, 8H, 4 × NCH_2_), 4.7.0 (s, 2H, SCH_2_), 5.51 (s, 2H, CH_2_CO), 7.12–7.15 (m, 2H, Ar-H), 7.47–7.53 (m, 2H, Ar-H), 7.59 (d, 1H, *J* = 8 Hz, Ar-H), 7.80 (d, 1H, *J* = 8 Hz, Ar-H), 7.90 (d, 1H, *J* = 8 Hz, Ar-H), 8.00–8.05 (m, 2H, Ar-H and CH-1,2,3-triazole), 12.60 (s, 1H, NH). ^13^C-NMR: δ 25.2 (SCH_2_); 41.2, 44.1, 48.2, 48.3, 51.4 (CH_2_); 111.2, 119.1, 121.6, 121.8, 122.2, 125.1, 126.3, 126.6, 126.9, 130.7, 135.6, 143.9, 150.2, 153.1, 164.8, 166.6, 168.8 (Ar-C, C=N, C=O) ppm. EI-MS (*m*/*z*): 490.13 (M^+^). Anal. Calcd for C_23_H_22_N_8_OS_2_: C, 56.31; H, 4.52; N, 22.84. Found: C 56.43; H 4.46; N 22.77.

#### 3.1.23. Characterization of 1-((1-(2-(4-(Benzo[*d*]thiazol-2-yl)piperazin-1-yl)-2-oxoethyl)-1*H*-1,2,3-triazol-4-yl)methyl)indoline-2,3-dione (**5l**)

White pellets, 89%, m.p. 246–248 °C. IR (*υ*, cm^−1^): 1570 (C=C), 1620 (C=N), 1705 (C=O), 2935 (C-H al), 3065 (C-H ar). ^1^H-NMR: δ 3.60–3.68 (m, 8H, 4 × NCH_2_), 5.00 (s, 2H, NCH_2_), 5.53 (s, 2H, CH_2_CO), 7.08–721 (m, 3H, Ar-H), 7.30 (t, 1H, *J* = 8 Hz, Ar-H), 7.50 (d, 1H, *J* = 8 Hz, Ar-H), 7.59 (d, 1H, *J* = 8 Hz, Ar-H), 7.63 (t, 1H, *J* = 8 Hz, Ar-H), 7.81 (d, 1H, *J* = 8 Hz, Ar-H), 8.09 (s, 1H, CH-1,2,3-triazole). ^13^C-NMR: δ 35.5 (SCH_2_); 41.3, 44.0, 48.0, 48.1, 51.2 (CH_2_); 111.7, 118.0, 119.2, 121.7, 121.9, 123.8, 124.9, 125.7, 126.5, 130.8, 138.5, 141.6, 150.6, 152.7, 158.3, 164.9, 168.5, 183.5 (Ar-C, C=N, C=O) ppm. EI-MS (*m*/*z*): 487.14 (M^+^). Anal. Calcd for C_24_H_21_N_7_O_3_S: C 59.13; H 4.34; N 20.11. Found: C 59.31; H 4.39; N 20.23.

#### 3.1.24. General Procedure for the Synthesis of 1,4,5-Trisubstituted-1,2,3-triazoles (**6a**,**b**)

Dimethyl/ethyl acetylenedicarboxylate (2 mmol) and benzothiazole azide (1 mmol) were heated in a water bath for 3 min. The reaction mixture was cooled, and then, ether was added to precipitate the product. The solid was filtered and washed with ether to obtain the desired product.

#### 3.1.25. Characterization of Dimethyl 1-(2-(4-(Benzo[*d*]thiazol-2-yl)piperazin-1-yl)-2-oxoethyl)-1*H*-1,2,3-triazole-4,5-dicarboxylate (**6a**)

Colorless needles, 94%, m.p. 176–178 °C. IR (*υ*, cm^−1^): 1570 (C=C), 1605 (C=N), 1715 (C=O), 2920 (C-H al), 3045 (C-H ar). ^1^H-NMR (400 MHz, CDCl_3_): δ 3.76–3.89 (m, 8H, 4 × NCH_2_), 3.97 (s, 3H, OCH_3_), 4.00 (s, 3H, OCH_3_), 5.63 (s, 2H, CH_2_CO), 7.15 (t, 1H, *J* = 8 Hz, Ar-H), 7.35 (t, 1H, *J* = 8 Hz, Ar-H), 7.62 (d, 1H, *J* = 8 Hz, Ar-H), 7.67 (d, 1H, *J* = 8 Hz, Ar-H). ^13^C-NMR: δ 41.8, 44.4, 47.7, 48.2, 51.4 (CH_2_); 52.7, 53.4 (2 × OCH_3_); 119.5, 120.9, 122.1, 126.3, 130.6, 130.8, 139.9, 152.3, 159.2, 160.3, 162.8, 168.2 (Ar-C, C=N, C=O) ppm. EI-MS (*m*/*z*): 444.12 (M^+^). Anal. Calcd for C_19_H_20_N_6_O_5_S: C 51.34; H 4.54; N 18.91. Found: C 51.42; H 4.49; N 18.99.

#### 3.1.26. Characterization of Diethyl 1-(2-(4-(Benzo[*d*]thiazol-2-yl)piperazin-1-yl)-2-oxoethyl)-1*H*-1,2,3-triazole-4,5-dicarboxylate (**6b**)

Colorless needles, 92%, m.p. 189–190 °C. IR (*υ*, cm^−1^): 1575 (C=C), 1610 (C=N), 1710 (C=O), 2935 (C-H al), 3040 (C-H ar). ^1^H-NMR: δ 1.24–1.33 (m, 6H, 2 × CH_3_), 3.61–3.73 (m, 8H, 4 × NCH_2_), 4.29–4.39 (m, 4H, 2 × OCH_2_), 5.83 (s, 2H, CH_2_CO), 7.11 (t, 1H, *J* = 8 Hz, Ar-H), 7.31 (t, 1H, *J* = 8 Hz, Ar-H), 7.52 (d, 1H, *J* = 8 Hz, Ar-H), 7.82 (d, 1H, *J* = 8 Hz, Ar-H). ^13^C-NMR: δ 14.1, 14.4 (2 × CH_3_); 41.5, 44.0, 48.0, 48.2, 52.3 (CH_2_); 62.0, 62.8 (2 × OCH_2_); 119.2, 121.7, 121.9, 126.5, 130.8, 130.9, 140.1, 152.7, 157.8, 160.5, 164.0, 168.5 (Ar-C, C=N, C=O) ppm. EI-MS (*m*/*z*): 472.15 (M^+^). Anal. Calcd for C_21_H_24_N_6_O_5_S: C 53.38; H 5.12; N 17.79. Found: C 53.22; H 5.20; N 17.66.

### 3.2. Anticancer Activity

#### Cell Proliferation Assay

Logarithmically proliferating cells were trypsinized, washed with PBS, and then transferred to fresh cultured medium. Cells were counted, plated into 96-well culture plates at a density of 1 × 10^4^ cell/ well, and kept in incubator (Binder, Tuttlingen, Germany) for 24 h to allow for adhesion. All cell lines were cultured in medium with 10% FBS and 100 U/mL penicillin and 0.1 mg/mL streptomycin at 37 °C in an atmosphere containing 5% CO_2_. Stock solutions (1 mM) of the synthesized compounds were prepared freshly prior to the experiment in dimethyl sulfoxide (DMSO) and serial dilutions were carried out to prepare concentrations ranging from 300–1 µM. The maximum DMSO concentration in the medium (0.1%) did not exhibit any significant effect on cell viability. Cells were incubated with the examined compounds for 48 h. Control wells were treated with 0.1% DMSO in medium or Doxorubicin as a standard anticancer agent. After an incubation time of 48 h, media was aspirated, and wells were washed with 200 µL of PBS. Then, 100 µL of freshly prepared MTT reagent was added to each well and incubated at 37 °C for 4 h. Afterward, the supernatant was aspirated, and 100% DMSO was added to solubilize the formed formazan crystals. The optical density (O.D) was obtained by reading the absorbance on ELISA plate reader (Palo Alto, CA, USA) at 540 nm and 670 nm. Cell survival percentages were plotted against the examined compound concentrations and IC_50_ values were determined. MCF7, T47D, HCT116, and Caco2 human cell cancer lines were used in this study. Each dilution of examined compound was tested in triplicate and IC_50_ values, i.e., compound concentration resulted in 50% inhibition of cell proliferation, was calculated based on the mean value of triplicate readings.

## 4. Conclusions

In the present work, we have designed and synthesized a series of novel potential anticancer agents based benzothiazole-piperazine-1,4-disubstituted-1,2,3-triazole molecular hybrids utilizing the Cu(I)-catalyzed 1,3-dipolar cycloaddition coupling between the appropriate 2-azido-1-(4-(benzo[*d*]thiazol-2-yl)piperazin-1-yl)ethanone with different functionalized and/or heterocyclic terminal alkynes. On the other hand, novel 4,5-diester-1,2,3-triazoles were synthesized using an efficient and quick green free solvent click synthesis in the absence of the copper catalyst. The synthesized compounds were evaluated against four different human cancer cell lines representing breast and colon cancers. Majority of the hybrid molecules displayed substantial antiproliferative activity. Among them, compound **5b** exhibited the most potent antiproliferative activity against all examined cancer lines. Preliminary in vitro screening showed that all compounds exerted good biological profile, which was further confirmed by clog*P* and ADME analysis. ADME and clog*P* analysis were in good agreement and follow Lipinski rule of five and violation rule.

## Figures and Tables

**Table 1:** Growth inhibition of T47D, MCF7, HCT116 and Caco2 human cancer cell lines after 48 h exposure time. IC_50_ values are expressed as mean ± SD of three independent experiments.

Code	T47D IC_50_ µM	MCF7 IC_50_ µM	HCT116 IC_50_ µM	Caco2 IC_50_ µM
**2**	>200	>200	>200	>200
**3**	162 ± 9	168 ± 11	185 ± 11	188 ± 10
**5a**	132 ± 6	128 ± 5	154 ± 8	145 ± 9
**5b**	38 ± 2	33 ± 4	48 ± 4	42 ± 2
**5c**	56 ± 7	61 ± 2	78 ± 9	68 ± 5
**5d**	44 ± 6	42 ± 3	51 ± 4	53 ± 3
**5e**	91 ± 8	88 ± 9	110 ± 6	103 ± 5
**5f**	164 ± 13	173 ± 12	184 ± 18	166 ± 9
**5g**	58 ± 8	55 ± 4	76 ± 5	72 ± 8
**5h**	48 ± 4	49 ± 2	69 ± 8	62 ± 8
**5i**	159 ± 9	168 ± 7	177 ± 11	182 ± 12
**5j**	95 ± 3	99 ± 7	102 ± 13	98 ± 6
**5k**	163 ± 10	160 ± 10	182 ± 17	178 ± 10
**5l**	125 ± 14	138 ± 16	145 ± 15	159 ± 11
**6a**	>200	>200	>200	>200
**6b**	161 ± 8	172 ± 10	176 ± 18	178 ± 7

Reference compound, Doxorubicin, IC_50_ values range between 1–10 µM against the examined different cell lines.

**Table 2:** In silico log*P* and ADME analysis.

Compounds	MW	log*P*	tPSA	nON	nOHNH	Vio	MV
**2**	340.25	2.55	36.44	4	0	0	250.19
**3**	302.36	2.51	86.19	7	0	0	257.20
**5a**	358.43	0.93	87.39	8	1	0	305.57
**5b**	386.48	1.41	87.39	8	1	0	339.19
**5c**	434.52	2.51	87.39	8	1	0	377.09
**5d**	510.62	4.17	87.39	8	1	1	448.11
**5e**	400.46	1.80	93.46	9	0	0	342.10
**5f**	531.67	2.99	97.88	10	0	1	452.13
**5g**	531.67	3.74	97.88	10	0	1	452.13
**5h**	545.70	4.12	97.88	10	0	1	468.94
**5i**	593.74	5.01	97.88	10	0	2	506.98
**5j**	507.67	4.34	80.05	8	0	1	417.66
**5k**	490.62	3.59	95.84	9	1	0	411.93
**5l**	487.55	2.14	106.23	10	0	0	408.89
**6a**	444.47	1.49	119.77	11	0	1	369.83
**6b**	472.53	2.24	119.77	11	0	1	403.43
Doxorubicin	543.52	0.57	206.08	12	7	3	459.18

MW: Molecular weight; log*P*: log octanol/water partition coefficient; tPSA: Total Polar Surface Area; nON: number of Hydrogen acceptors; nOHNH: number of Hydrogen donors and MV: Molecular Volume; Vio: Violation number Lipinski’s rule.
